# Effectiveness of technology-enhanced learning in neuroanatomy education among health professionals - a systematic review

**DOI:** 10.1186/s12909-026-09764-w

**Published:** 2026-06-23

**Authors:** Anniesmitha K, Mohandas Rao K G, Ashwini Aithal P, Nikita N. Bandekar, Tatiyana Mandal, Bincy M. George

**Affiliations:** 1 Division of Anatomy, Department of Basic Medical Sciences (DBMS), Manipal Academy of Higher Education, Manipal, 576104 India; 2 Division of Pharmacology, Department of Basic Medical Sciences (DBMS), Manipal Academy of Higher Education, Manipal, 576104 India

**Keywords:** Technology-enhanced learning, Neuroanatomy, 3D visualization, Anatomy, Effectiveness

## Abstract

**Background:**

Neuroanatomy is widely recognised as one of the most challenging subjects in health professions education due to its complex three-dimensional structures and spatial relationships. Technology-enhanced learning has increasingly been incorporated into neuroanatomy education to facilitate learning. This review aimed to evaluate the effectiveness of technology-enhanced learning interventions in teaching practical neuroanatomy skills.

**Methods:**

This review was conducted in accordance with the Preferred Reporting Items for Systematic Reviews and Meta-Analyses (PRISMA) guidelines. Randomised controlled trials published between 2013 –2023 that evaluated technology-enhanced learning interventions in neuroanatomy education were included using a Rayann software. Nine studies met the eligibility criteria and were analysed using the modified Kirkpatrick model to assess educational outcomes.

**Results:**

Learner’s motivation and engagement (Kirkpatrick Level 1) were consistently high. for virtual reality,3D visualisation, and stereoscopic learning modules, with students reporting increased satisfaction, confidence, and perceived understanding. Augmented reality inventions demonstrated more valuable responses, often influenced by design, quality, and implementation. Improvements in objective learning outcomes (Kirkpatrick Level 2) were reported in several studies, particularly those employing 3D visualisation and virtual reality tools. However, these findings were not consistent across all interventions. Technology enhanced learning appeared particularly beneficial for supporting cognitive engagement and visuospatial understanding of complex neuroanatomical concepts. Considerable heterogeneity was observed across intervention types, pedagogical approaches, and outcome measures. Few studies evaluated behavioural changes (Kirkpatrick Level 3) or long term educational impact (Kirkpatrick level 4).

**Conclusion:**

Technology -enhanced learning, particularly 3D visualisation and virtual reality, shows promise as a complementary approach to neuroanatomy education by enhancing learner’s engagement, motivation, confidence, and spatial understanding. While improvement in object knowledge, outcomes were reported in several studies, evidence of superiority over conventional teaching approaches remains inconsistent. The evidence base was restricted to randomised controlled trials and was driven from undergraduate medical student populations, which may limit the generalisability of the findings. Further high -quality longitudinal studies are needed to evaluate behavioural change, long-term knowledge retention, and educational impact.

**Supplementary Information:**

The online version contains supplementary material available at https://doi.org/10.1186/s12909-026-09764-w.

## Introduction

Technology-enhanced learning (TL) emerged as a transformative approach in neuroanatomy education for providing flexibility and broader accessibility to students and educators, especially considering challenges posed by the COVID-19 pandemic [1, 2]. 3D visualisation tools, virtual reality (VR), and augmented reality (AR) are advancements that offer interactive learning experiences that surpass the limitations of traditional methods like physical models and textbooks and enable students to explore spatial relationships between anatomical structures more effectively [3–10]. These tools are particularly valuable in regions with limited access to cadaveric dissection facilities, providing an alternative for teaching complex neuroanatomical concepts [11–13].

Despite innovations, the effectiveness of TL in developing practical skills like dissection and neuroanatomical identification remains debated with studies showing improved theoretical knowledge [14], but required further exploration. Traditional methods combining theory and practice [11, 15–17], face challenges due to reduced teaching hours [11, 16, 18], raising concerns about TL's ability to fully replace them [19].

In the realm of medical education, contemporary pedagogical approaches increasingly emphasise aligning with this principle and transforming traditional medical curricula [20], Studies demonstrate that technology-based interventions, including serious games and social collaborative VR environments, can foster a higher learning outcomes in anatomy and related fields [21, 22]. Longitudinal investigation further suggests that these immersive technologies can contribute to enhanced knowledge retention and the development of essential collaborative skills, crucial for future healthcare professionals [23]. Integrating these innovative tools into medical education represents a significant opportunity to create more effective and engaging learning experiences, better preparing students for the challenges of modern healthcare practice.

The aim of this review is to answer the efficacy of technology-enhanced learning (TL) resources in assisting the development of practical neuroanatomical skills, with a focus on anatomical identification and dissection, by focusing on randomised controlled trials (RCTs) and assisting the study with Kirkpatrick model will provide the highest level of evidence in evaluating the effectiveness of intervention [24, 25]. Though TL presents promising opportunities for the future of neuroanatomy education, especially in terms of accessibility and flexibility, its effectiveness in teaching practical skills remains under question. By following a proper systematic review guideline, the review will critically examine the role of digital platforms in fostering these skills and assess their potential to enhance neuroanatomy education methods.

## Methodology

### Protocol registration and reporting

This review is reported as per the Preferred Reporting Items for Systematic Reviews in the International Prospective Register of Systematic Reviews (PROSPERO) database, and its registration number is CRD42024569747.

### Data sources and literature search

A comprehensive literature search was conducted in five databases, i.e., Scopus, PubMed MEDLINE, EBSCO, The Cochrane Library, and Web of Science, with the Boolean operators ‘AND’ and 'OR', the detailed search string is attached to this manuscript. The search was performed with restrictions placed on English-language publications and a publication period from 2013 to 2023. Searches were re-run just before final analysis to capture any additional studies.

### Eligibility criteria

The inclusion criterion for this review was that it used a randomised control trial study design with full-text articles in English, and the articles were published from 2013–2023.

The exclusion criteria were as follows: studies with insufficient details regarding the inclusion criteria, such as studies other than RCTs; review studies and letters to editors; studies that examined the effectiveness in other subjects; studies that did not assess effectiveness or satisfaction; and studies conducted in other medical subjects.

Health science (medical, allied health) and excluding studies on veterinary science and other non-health science disciplines (e.g., students or professionals outside medical, allied health, or neuroscience-related fields). Technology-enhanced learning methods (e.g., using only printed materials, textbooks, or purely text-based content) or interventions without any interactive or enhanced component (e.g., plain 2D images without interactivity).

### Selection process

Selection and data extraction of identified studies were first screened by title and abstract, followed by full-text screening based on pre-defined inclusion and exclusion criteria using Rayyan (software). The extraction of data was done using a comprehensive form, which was created and reviewed by the team. On a consensus basis, modifications were made, and a final data sheet was created in Microsoft Excel to collect information such as author and year of publication, population, sample size, study design and sampling method, intervention details, duration added, subject covered, and participant learning outcomes. Two reviewers individually and independently performed selection process and data extraction. All the disagreements were resolved by consensus or through consultation with a third reviewer.

The methodological quality of the included studies was assessed using the Joanna Briggs Critical Appraisal (JBI) checklist for randomized controlled trials (RCTs) [26], which evaluates the randomization of participants, concealment of allocation to treatment groups, similarity of treatment groups at baseline blinding of participants, delivering treatment, outcome assessors, follow-up of their completeness, analysis according to group assignment, consistency in outcome measurement, reliability of outcome measurement, appropriateness of statistical analysis, suitability of trial design, and handling of deviations from standard RCT design, responses to the checklist were categorized with "yes" or "no" in alignment with the question prompt, followed by calculation and analysis of "yes" responses.

The extracted data were analysed using narrative summaries and were represented in appropriate graphs.

## Findings

**Fig. 1:**
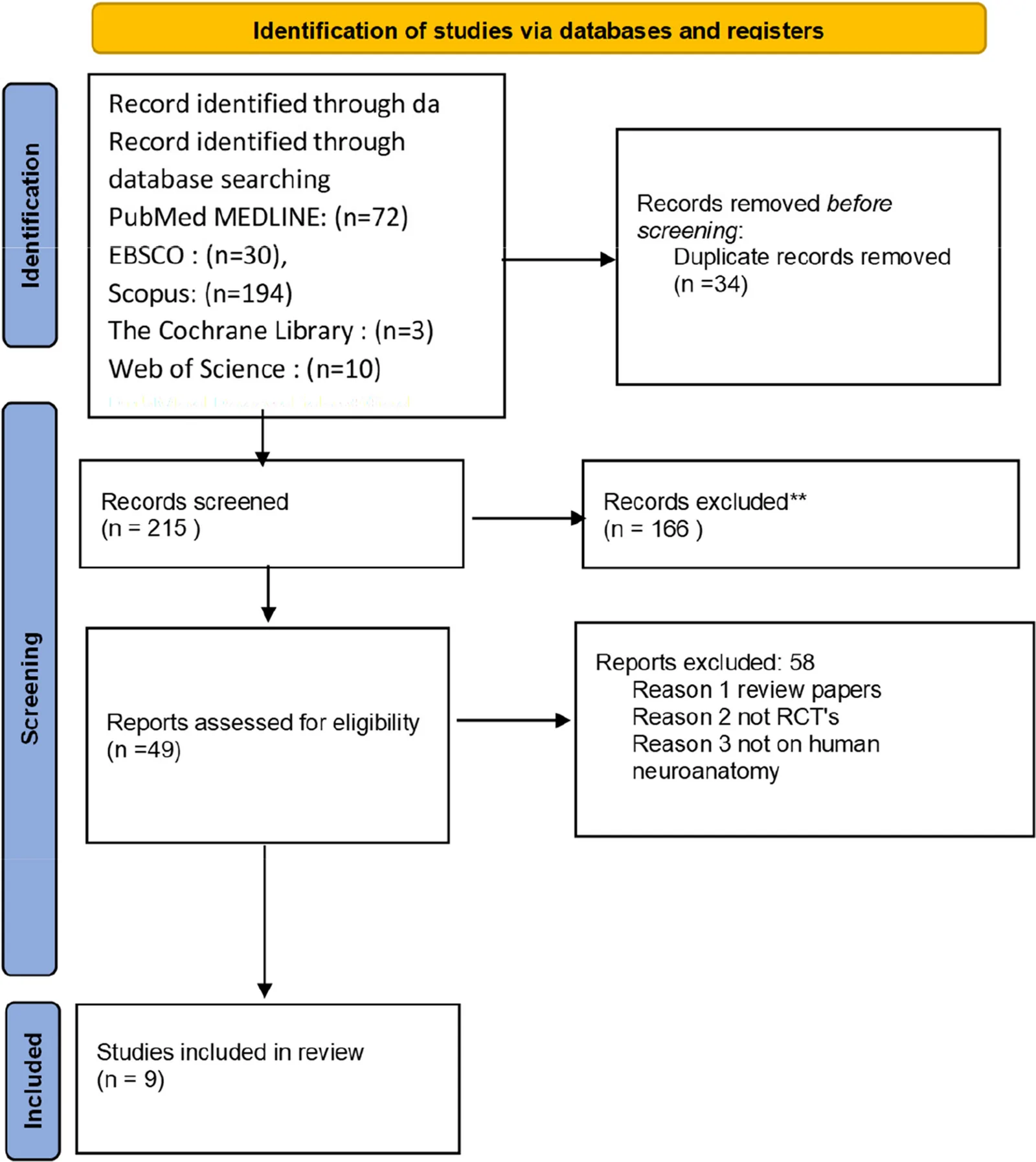
Prizma chart. Page MJ, McKenzie JE, Bossuyt PM, Boutron I, Hoffmann TC, Mulrow CD, et al. The PRISMA 2020 statement: an updated guideline for reporting systematic reviews. BMJ 2021;372:n71. https://doi.org/10.1136/bmj.n71. For more information, visit: http://www.prisma-statement.org/

The screening process conducted through a systematic approach [27] is shown in Fig. 1, with nine included articles, which is summarised in Table 1.

**Table 1:** Characteristics of included studies. The table summarizes key information such as author, year, country, participant age, population type, sample size, intervention details, and primary and secondary outcomes. Primary outcomes primarily focus on post-test scores, while secondary outcomes highlight student satisfaction and engagement levels. Studies showed significant improvements in knowledge retention and practical skills when students were exposed to 3D technologies and online interventions compared to traditional methods. NM: Not mentioned

Sl.no	Author	Year/ Country	Age range	Participant ands Sample Size	Intervention	Outcome measure	Primary Outcome (Post-intervention -knowledge (Test Scores)	Secondary Outcome (Satisfaction & Engagement)
1	Shao et al	2015 China	NM	Clinical students 60	3D Visualization	Subjective questionnaire analysis of three groups of students	Post-test: Hand-held scanner 3D imaging group: 76.6 ± 5.2, 2D fitting 3D method group: 85.5 ± 5.3	Clinical practice and teaching satisfaction significantly higher in 3D groups (p < 0.001)
2	Kockro et al	2015 Switzerland	20.6	2nd year Medical students 169	Virtual reality	10 multiple-choice questions (MCQs) relating to the topographical anatomy of the third ventricle	3D presentation was statistically non-inferior to 2D (p < 0.0001); no significant difference between mean scores (p = 0.215)	Students rated 3D superior to 2D in spatial understanding, application, and enjoyment (p < 0.01)
3	Stepan et al	2017 New York	20–30	1 st and 2nd year Medical students 66	Augmented reality	Mixed question type anatomy tests immediately before, immediately after and 8-weeks after a 30-min laboratory	No significant difference in pre-post improvement; retention quiz: VR = 0.68 ± 0.25, Control = 0.64 ± 0.23	VR group found learning more engaging and scored higher on motivation assessment (p < 0.01)
4	Faria et al	2023 Uberlândia	21–25	Medical graduate students 84	3D interactive stereoscope	A written theory test and a lab practicum	Group 1 (stereoscopic) outperformed control in both theoretical and practical exams (p < 0.05)	NM
5	Drakin et al	2015 Utah	NM	1 st Medical students 73	Virtual neuroanatomy lab	32 identification question exam on MRI –survey of satisfaction and self ratedconfidence level in neuroanatomy	Significant improvement in C-shaped structure recognition in 3D group (p < 0.01)	100% of students in the 3D group recommended the tool for future use
6	Allen et al	2016 Canada	23.6	2nd Medical students 47	Augmented learning tools	-question knowledge assessment of spatial relationships in neuroanatomy	Group A (3D) performed significantly better than Group B (cadaveric) in knowledge assessments (p < 0.01)	Majority of participants supported development of similar 3D resources for the curriculum (Likert scale: 4.03/5)
7	Henssen et al	2020 Netherlands	NM	1 st Medical students and Biomedical science 31	Online 3D teaching methods	Questionaries on related modules,	Control group outperformed AR group on post-test scores; no significant difference in cognitive load	Focus group: AR users were less satisfied compared to traditional learners
8	Mendez-Lopez et al	2022 Spain	19.2 ± 1.7	neurophsychology students 67	3D imaging	A questionnaire to assess students’ knowledge of neuroanatomy was designed using eight questions	2D method outperformed AR in post-test results, but both groups improved	AR was highly valued by students for engagement, and no motivational barriers were reported
9	Bernard et al	2020 France	20.2	2nd year medical students 190	Interactive 3D stereoscopy	30-mixed question type anatomy tests immediately before and 1 month after 5- min laboratory	3D group significantly outperformed 2D group in knowledge retention, especially in anatomical relationships (p < 0.01)	82.5% of students enjoyed using stereoscopic video; 85% supported it as a complementary tool

There were two articles published in the United States of America (USA) and one each in China, Switzerland, Brazil, Canada, the Netherlands, Spain, and France. Participants of these articles included undergraduate medical students in all articles except one article that involved neuropsychology students and another with bioscience students along with medical students. The student's age range was between 19 and 25 years, as the study was conducted among 1st- and 2nd year medical students, which is listed in Table 1. The interventions across studies utilised various forms of learning methods in which four articles used 3D visualisation [3, 5, 8, 28], one in virtual reality [29]; two articles with interactive stereoscopic online lectures [4, 30] and two articles with augmented reality [6, 7]. These interventions were specifically designed to enhance the student’s ability to visualise complex anatomical structures, making them suitable for subjects like neuroanatomy, which often involves complex spatial understandings. Eight studies compared traditional methods such as didactic lectures and cadaveric dissection with technology-enhanced learning interventions [3–8, 29, 30] and one article with a 2D stereoscopic image [28]. In general, the control groups in these studies were exposed to traditional neuroanatomy educational environments (Table 1). All nine studies measured post-intervention knowledge test scores as their primary outcome. These scores reflected mixed ability to understand and retain neuroanatomical knowledge after undergoing technology-enhanced interventions. Across all studies, those who used 3D visualisation and interactive technology-enhanced methods demonstrated improved test performance compared to those who engaged in traditional learning environments. Several studies also gathered qualitative data through surveys, examining student satisfaction with the teaching methods. The interactive elements, particularly 3D visualisations, were praised for being more engaging and user-friendly, enhancing both understanding and retention of complex anatomical concepts. The JBI risk of bias checklist was used to assess several dimensions of study quality. The studies demonstrated moderate to high quality, with risk of bias scores ranging from 7–9 out of 13, as detailed in Table 2.

**Table 2:** JBI Risk of Bias Checklist assessment (Q1-Q13). The table assesses study quality using the JBI checklist, with questions covering key domains such as randomization (Q1), allocation concealment (Q2), baseline group similarity (Q3), blinding of participants (Q4), blinding of those delivering treatment (Q5), blinding of outcome assessors (Q6), identical treatment of groups except for the intervention (Q7), completeness of follow-up (Q8), analysis of participants in the groups they were randomized to (Q9), consistency in outcome measurement (Q10), reliability of outcome measures (Q11), appropriateness of statistical analysis (Q12), and suitability of trial design (Q13). The studies are rated based on these criteria, with scores reflecting moderate to high methodological quality

Record no	Author	Q1	Q2	Q3	Q4	Q5	Q6	Q7	Q8	Q9	Q10	Q12	Q13	Overal apprical
1	Xuefei Shao et al	Yes	Unclear	Yes	No	no	Unclear	Yes	unclear	Yes	Yes	Yes	Yes	7/13
2	Ralf A. Kockro E et al	Yes	Unclear	Yes	No	No	Yes	Yes	Yes	Yes	Yes	Yes	Yes	9/13
3	Katelyn Stepan et al	Yes	Unclear	Yes	No	Unclear	Unclear	Yes	Yes	Yes	Yes	Yes	Yes	8/13
4	Jose Weber et al	Yes	Unclear	Yes	No	No	Unclear	Yes	Yes	Yes	Yes	Yes	Yes	8/13
5	Zachany A Drakin et al	Yes	Unclear	Yes	Yes	No	Unclear	Yes	Yes	Yes	Yes	Yes	Yes	9/13
6	Allen et al	Yes	Unclear	Yes	No	Unclear	Unclear	Yes	Yes	Yes	Yes	Yes	Yes	8/13
7	Lopez et al	Unclear	No	Yes	No	No	No	Yes	Yes	Yes	Yes	Yes	Yes	7/13
8	Bernard et al	Yes	No	Yes	No	NA	Unclear	Yes	No	Yes	Yes	Yes	Yes	6/13
9	Henssen et al	Yes	Unclear	Yes	No	No	Unclear	Yes	Yes	Yes	Yes	Yes	Yes	8/13

### 3D visualisation modules

Allen et al. (2016) assessed a 3D neuroanatomical e-learning module and the connection between students' neuroanatomical knowledge and spatial skills. The participants in the crossover trial were split into two groups and given access to either the gross anatomy lab or the 3D online learning module after completing tests measuring their spatial and anatomical knowledge. Prior to using the other learning modality, the participants finished a second exam to test their knowledge. The participants in both groups performed better on Quiz 1 than on the pretest knowledge evaluation. Students who first used 3D online resources performed much better on Quiz 1 than those who used gross anatomy resources did. The Quiz 2 scores improved significantly for participants who accessed the 3D learning module after exposure to cadaveric resources. After exposure to both learning modes, there were no significant differences between the groups. There were significant positive relationships between participants' spatial ability scores and their performance on the pretest, Quiz 1, and Quiz 2 assessments (*r* = 5, 0.22, *P* = 5, 0.04; *r* = 5, 0.25, *P* = 5, 0.02; *r* = 5, 0.26, *P* = 5 0.02). These preliminary findings revealed that students enjoyed working with the 3D e-learning module and that their learning outcomes improved significantly after they used the resource.

Shao et al. (2023) examined the effectiveness of two-dimensional fitting and three-dimensional imaging approaches in neuroanatomical instruction. Twenty clinical students in each of the three groups—traditional instruction, handheld scanner 3D imaging, and 2D fitting 3D methods—were randomly selected from among 60 clinical students in 2020 at Wannan Medical College. Following an analysis and comparison of the modelling images from the 2D fitting 3D method group and the hand-held scanner 3D imaging group, the teaching outcomes of the three groups are assessed via both objective and subjective evaluation techniques. Exam papers, unified propositions, and unified scores serve as objective evaluations, whereas questionnaires are used for subjective evaluations. In terms of exam outcomes, clinical practice assessment, and teacher satisfaction, the hand-held scanner 3D imaging group outperformed the traditional teaching group (*P* < 0.01).

### Virtual reality

Stepen et al., Human Digital Imaging and Communications in Medicine (DICOM) computed tomography (CT) imaging and magnetic resonance imaging (MRI) were built into 3D virtual reality (VR) formats united with a head-mounted display with tracking abilities, allowing for an immersive VR experience. A focused interactive module was created that highlights the ventricular system and cerebral vasculature. A randomised controlled study was conducted with 66 medical students, 33 each in both the control and experimental groups. The control group was given an online textbook, and the VR interactive model was given to the intervention group to study neuroanatomical structures. They evaluated the students’ anatomy knowledge, educational experience, and motivation (using the Instructional Materials Motivation Survey [IMMS], a previously validated assessment) and reported that there was no significant difference in anatomy knowledge between the 2 groups in terms of preintervention, postintervention, or retention quizzes. The VR group reported that the learning experience was significantly more engaging, enjoyable, and useful (all *p* < 0.01), scored significantly higher on the motivation assessment (*p* < 0.01), and concluded that the technology was as effective as traditional textbooks in teaching neuroanatomy.

Kockro et al., 2015: Medical students in their second year (*n* = 169) were randomly assigned to attend a standardised audio lecture on the anatomy of the third ventricle, which was prerecorded, and a two-dimensional (2D) PowerPoint presentation (*n* = 80) was given. Immediately following the lecture, the students took a 10-question multiple-choice exam on the topic covered as well as a subjective evaluation of the teaching methods. The 2D group had a mean score of 5.19 (± 2.12), whereas the 3D group had a mean score of 5.45 (± 2.16). The findings of the 3D group were not significantly inferior to those of the 2D group (*p* < 0.0001). The students concluded that stereoscopically enhanced 3D lectures are effective ways to teach neuroanatomical knowledge and are well received by students [31]. They rated the 3D method better than 2D teaching in four domains (spatial understanding, application in future anatomy classes, effectiveness, and enjoyableness) (*p* < 0.01).

De Faria et al., 2016 A manual turntable was used to take pictures of the relevant parts of 40 fresh brains (each with 80 hemispheres), which were then processed and saved in a database with 5337 photos. Three groups—one (traditional technique), two (interactive, not stereoscopic), and three (interactive, stereoscopic)—were used to conduct a pedagogical evaluation with 84 graduate medical students. Both a written theoretical test and a lab practicum were used to assess the approach. Groups 2 and 3 were different from Group 1 (*p* < 0.05) and had the highest mean scores in the pedagogic assessments. There was no significant difference between Groups 2 and 3 (*p* > 0.05). Size effects, determined by comparing scores before and after lectures, show the effectiveness of the approach. The ANOVA results revealed no significant difference between Group 2 and Group 3 in the practicum. Nevertheless, these two groups were significantly different from Group 1 (*p* < 0.05). Compared with typical teaching tools, the authors determined that this strategy further enhanced student knowledge and increased learning [32–40].

Drapkin and his team in 2015 has generated a three-dimensional (3D) computerised teaching tool in general virtaual reality for neuroanatomy that was developed to train medical students to appreciate subcortical structures on a magnetic resonance imaging (MRI) series of the human brain. The effectiveness of the tool was evaluated by comparing the results of an MRI identification quiz and a survey between two groups of first-year medical students. The first group was taught with a new, innovative 3D teaching tool, whereas the second group was taught with conventional teaching tools, such as 3D models from widely used textbooks or online resources with similar guidelines. The students in the experimental group performed better than did those in the control group did; moreover, 100% of the students in the experimental group recommended this tool for other students. According to the findings, the neuroanatomy teaching tool trains germ physicians in reading a mind MRI and performs notably well in teaching C-shaped inside-mind areas [41, 42].

### Interactive stereoscopic online lectures:

Bernard et al., 2020: The University of Angers carried out a prospective controlled investigation at a single site. After an assessment of content understanding, an educational video on neuroanatomy was presented. After watching the film in either 2D or 3D, the participants took a written test on anatomy. Independent t tests on total score, basic anatomical knowledge, anatomical correlations, and reasoning were used to assess pre- and post-video performance. According to the results, 175 participants finished the study at baseline, and the 2D (*n* = 86) and 3D (*n* = 91) groups were comparable in terms of age and class level. In both the pre-test and the post-test on fundamental knowledge, the 3D and 2D scores were comparable (mean 73.2% vs. 74.4%, *p* = 0.37). In terms of anatomical linkages (mean 86.4% vs. 63.5%, *p* = 0.004), clinical inference/reasoning (mean 76.8% vs. 67.6%, *p* = 0.023), and total notes (mean 76.8% vs. 67.6%, *p* = 0.07), the 3D group's average scores on the post-test were higher. According to 70 students (77%) who answered the 3D student satisfaction questionnaire (*n* = 91), the stereoscopic film made it possible to see anatomical structures in 3D. Using the stereoscopic video was enjoyable for the students (*n* = 75, 82.5%). Most students (*n* = 78, 85%) who supported the use of this type of stereoscopic 3D video in their regular teaching as a complementary tool agreed that incorporating 3D videos as ancillary teaching into curricula would improve students' understanding of anatomical relationships [4].

### Augmented reality

Henssen et al. (2020) used AR applications or brain cross-sections to examine the effects of neuroanatomical learning on test results, cognitive load, and motivation. Two practical tasks are preceded by a mental rotation test (MRT) and a pre-test consisting of extended matching questions, double-choice questions, and a test on cross-sectional anatomy. Students in the two groups were categorised based on their MRT scores. Studying general brain architecture and subcortical regions was the goal of the two practical exercises. A motivational questionnaire and a post-test that was comparable to the pre-test were then completed by the subjects. Finally, a focus group interview was conducted to gauge members' opinions. The participants in this experiment were 31 medical and biomedical students, with a mean age of 19.2 ± 1.7 years, 19 of whom were male (61.3%) and 12 of whom were female (38.7%). Compared with the students with GreyMapp-AR, the students who worked with cross-sections (*n* = 16) improved their test scores much more (*P* = 0.035) (*n* = 15). The analysis revealed that this difference was due primarily to the significant improvement in the number of cross-sectional questions. Compared with students in the GreyMapp-AR group, those in the cross-sectional group also reported significantly greater relevance to the subject under consideration (*P* = 0.009) and extraneous cognitive load (*P* = 0.016). There were no significant variations in motivating scores. This study implies that AR apps can play a role in future anatomy teaching as add-on instructional tools, notably in learning three-dimensional relationships between anatomical parts [6].

Lopez et al., 2022: Technologies have been used to assist in anatomy learning by visualising anatomy content in three dimensions (3D). Augmented reality (AR) is a promising technique in this domain. A mobile AR application for visualising morphological and functional information of the brain was created. A sample of 67 neuropsychology students took exams to assess their visuo-spatial aptitude, anatomical knowledge, learning goals, and technological experience. Simultaneously, when participants conducted a learning task utilising one of the visualisation approaches (3D) via the AR application and a two-dimensional (2D) approach involving the use of a textbook to colour, they were asked questions about their pleasure and knowledge. The two methods boosted knowledge similarly, but the 3D method had greater satisfaction ratings and was more popular among students. The 3D technique was similarly preferred by the pupils who utilised it for the activity. After controlling for the approach employed in the activity, connections were discovered between the preference for the 3D method and experience with technology. The results showed that the pupils valued the AR application [7].

## Discussion

The review evaluates the effectiveness of technology-enhanced learning (TL) interventions in neuroanatomy education, emphasising randomised controlled trials (RCTs) to minimise bias. Table 3 presents the characteristics of the included studies, supporting the assertion that the findings provide robust evidence [25]. The nine included studies have shown how technologies have contributed to skill development and their impact on practical skills like dissections and anatomical identification.

**Table 3:** Characteristics of randomized controlled trial designs and approach to analysis (Q1-Q6). The table evaluates the inclusion of qualitative process evaluations (Q1), subgroup analyses (Q2), and evidence of intervention effects (Q3), alongside the presence of longitudinal components beyond immediate post-tests (Q4). It also reviews whether the analysis included limitations to generalization (Q5), and whether the studies referenced educational theory (Q6). Most studies demonstrated intervention effects, and many incorporated theory to discuss broader implications, (Q7) Studies conducted in multicentre

Author	. Characteristics of randomized controlled trial designs and approach to analysis*							
	Q1	Q2	Q3	Q4	Q4	Q5	Q6	Q7
Xuefei Shao et al	Yes	Unclear	Yes	No	Yes	Yes	Yes	No
Ralf A. Kockro E et al	No	Yes	Yes	No	Yes	Yes	Yes	No
Katelyn Stepan et al	No	Yes	Yes	Yes	Yes	Yes	Yes	No
Faria et al	No	Yes	Yes	No	Yes	Yes	Yes	No
Zachany A Drakin et al	No	Yes	Yes	No	Yes	Yes	Yes	No
Allen et al	No	No	Yes	No	Yes	Yes	Yes	No
Henssen et al	Yes	Yes	Yes	Yes	Yes	Yes	Yes	No
Lopez et al	No	Unclear	Yes	Yes	Yes	Yes	Yes	No
Bernard et al	No	No	Yes	Yes	Yes	Yes	Yes	No

Most of the participants were medical students at undergraduate and graduate levels, aged between 19 and 25 years, and one study examined psychology students. Most participants included in this study were undergraduate and graduate medical students, with only one study extending beyond medicine to include psychology students. Consequently, the current evidence base is highly centred on medical education, particularly at the undergraduate level. Limited evidence from other learner groups prevents firm conclusions regarding its effectiveness in nursing, dentistry, allied health professions, postgraduate training, or continuing professional education. While the educational benefits observed in medical students may be applicable to other health professions, this assumption has not been adequately tested. Therefore, the transferability of these findings beyond undergraduate medical education remains uncertain and requires further investigation in more diverse educational settings [34].

**Fig. 2:**
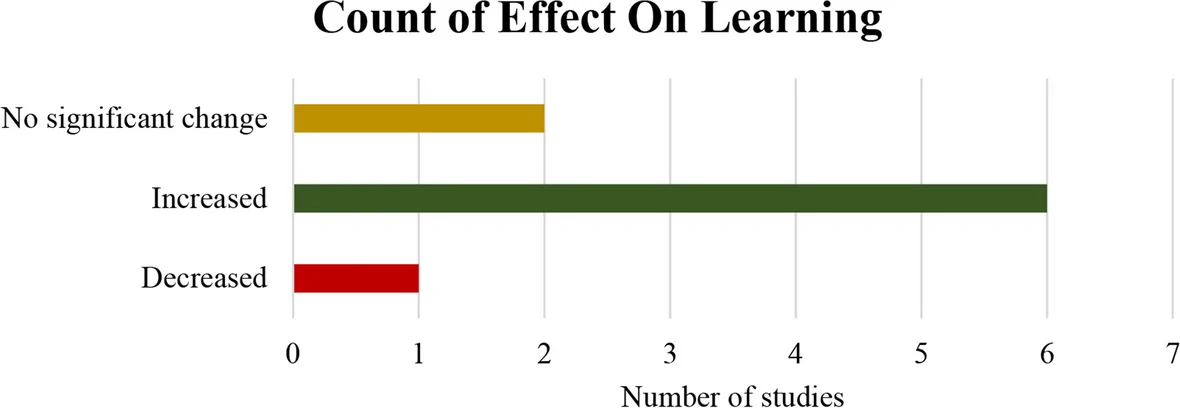
Effect on learning (Kirkpatrick Level 2) of technology-enhanced learning tools. The bar graph illustrates the effect on learning (Kirkpatrick Level 2) across studies, categorized by statistical significance: green indicates significant improvement (*p* < 0.05), yellow indicates no significant change (*p* > 0.05 or not mentioned), and indicates a significant decrease in learning outcomes

The effectiveness of technology-enhanced learning tools in neuroanatomical education, as summarised in Tables 4 and 5 was assessed via the Kirkpatrick model, which includes four levels: learner reactions (L1), learning (L2), behaviour (L3), and impact (L4). Notably, most studies have focused on L1 and L2, with minimal assessments of behaviour and impact (L3 & L4) and Fig. 2, illustrates the effect on learning (Kirkpatrick Level 2) across studies.

**Table 4:** Effectiveness assessed using Kirkpatrick’s Model—Level 2: Learning, Impact

First Author	Effect on Learning (L2)	P-value
Xuefei Shao et al	Increased	< 0.01
Ralf A. Kockro E et al	Increased	< 0.0001
Katelyn Stepan et al	Increased	< 0.01
Faria et al	Increased	< 0.05
Zachany A Drakin et al	No significant change	> 0.05
Allen et al	Increased	< 0.01
Henssen et al	Decreased	0.035
Lopez et al	No significant change	Not mentioned
Bernard et al	Increased	< 0.01

**Table 5:** Effectiveness assessed using Kirkpatrick’s Model—Level 1: Reaction, Level 2: Learning, Level 3: Behaviour, Level 4: Impact

First Author	Kirkpatrick's level			
	Level 1: Effect on reaction	Level 2: Effect on Learning	Level 3: Effect on Behaviour	Level 4: Effect on impact
Xuefei Shao et al	Subjective evaluations via post-course questionnaires showed high student satisfaction with both 3D imaging methods—especially the 2D-fitting approach	Learning increased: Significant improvement in exam scores (objective tests) in both 3D groups, with 2D-fitting > scanner > traditional (*P* < 0.01)	Clinical practice assessment scores mirrored the written‐test gains (handheld-scanner > traditional, and 2D-fitting > scanner; *P* < 0.01)	Not formally measured (authors note enhanced post-processing ease and broad application potential)
Ralf A. Kockro E et al	In all four evaluation domains, 3D VR performed better than traditional instruction relative to 2D presentations (Fisher's exact test; *p* < 0.001 in all cases): Four questions were posed to students to find out their individual thoughts regarding their educational experience	Learning Increased (*p* < 0.0001). Ten multiple choice questions (MCQs) pertaining to the third ventricle's topography	Not assessed	Not assessed
Katelyn Stepan et al	Higher likelihood of recommending the study material in VR group versus other students (*p* < 0.01). No difference between the 2 groups in their responses to the ease of use of the study materials: The Instructional Materials Motivation Survey (IMMS): 4 key aspects of motivation in education: attention, relevance, confidence, and satisfaction	Increased (*p* < 0.01): Three quizzes were administered: a pre-intervention quiz, a post-intervention quiz, and a retention quiz. The quizzes assessed knowledge of the ventricular system, arterial supply to the brain, and the brainstem using multiple choice and fill-in-the-blank questions. The post-intervention quiz also included identification questions based on a 3D brain model. The quizzes were developed by an otolaryngologist and a resident	Not assessed	Not assessed
Faria et al	Increased (Qualitative): Those who participated in the interactive sessions, with or without stereoscopic elements (Groups 2 and 3), were asked to comment on the efficacy and short comings of the instructional materials	Increased (*p* < 0.05): Pre-test: The students were asked to compile a list of the limbic system’s components. Pedagogical evaluations were performed after each class	Increased (*p* < 0.05): In the practical assessment, students identified anatomical structures on real specimens at 10 stations/	Not assessed
Zachany A Drakin et al	the interactive 3D multimedia module received higher satisfaction ratings from students (*P* < 0.05): survey of satisfaction & self rated confidence	No Statistically significant (*P* > 0.05): Pre-test: Previous experience survey .Purdue visualizations of Rotation test Post-test: 32 identification questions MRI	Not assessed	Not assessed
Allen et al	Qualitative feedback: students “appreciated” and “enjoyed” working with the 3D module	Learning increased: Students scored significantly higher after using 3D (P < 0.01)	Not assessed	Positive correlation between spatial ability and gains (r≈0.25); module may boost spatial skills and anatomy success
Henssen et al	No difference in overall motivational questionnaire scores between AR and cross-section groups	Learning decreased with AR: Cross-section group showed greater improvement in test scores (*P* = 0.035)	Cognitive‐load measures: cross-section group experienced higher germane (*P* = 0.009) and extraneous load (*P* = 0.016)	Not assessed
Lopez et al	AR students reported significantly greater satisfaction on both the Learning Experience Survey (LES: U = 277.5, *P* < 0.001) and Learning Method Survey (LMS: U = 334.0, *P* = 0.004)	No significant change: Both AR and 2D groups improved equally in knowledge tests	Students were 12 × more likely to say “I find it easier to learn with …” AR if they had used AR (odds = 12.3, χ^2^ = 12.3, P < 0.001)	Not assessed
Bernard et al	77% “agreed” stereoscopy gave good 3D views; 82.5% “enjoyed” it; 85% “supported” its routine use as a complement	Learning increased: Higher scores in spatial understanding and clinical reasoning in 3D group (*P* < 0.01)	Not assessed	Suggests improved spatial‐reasoning skills, but no follow-up on real-world impact

3D visualisation modules, as reported by Shao et al. and Allen et al., were found to be highly effective. Both studies demonstrated high learner satisfaction and statistically significant improvements in knowledge (p < 0.01). Shao et al. additionally reported enhanced clinical task performance, whereas Allen et al. noted a link between spatial ability and academic success, indicating potential for behavioural and long-term impact.

Virtual reality (VR) tools, which were examined in four studies, consistently increased learner motivation (L1) and improved learning outcomes in most cases. Faria et al. further evaluated behavioural application, showing better identification of anatomical structures during practical assessments (L3), although none of the VR studies addressed long-term impact (L4) [32–40, 43].

Interactive Stereoscopic learning, represented by Bernard et al., received positive learner feedback, and led to significantly better performance in spatial and clinical reasoning tasks (*p* < 0.01). However, follow-up at the behavioural and impact levels was lacking.

In the case of augmented reality (AR), the findings were mixed. While Lopez et al. reported high learner satisfaction and a strong preference for AR tools (L1), Henssen et al. reported no motivational or learning advantage [5, 44–46]. Allen et al. reported significant learning gains (*p* < 0.01) and was the only AR study to suggest a potential long-term benefit by linking AR-enhanced spatial ability with academic performance. These studies encouraged the areas of the anatomical curriculum in including the development of 3D learning resources [47–49] and more notably in neuroanatomy [38, 50].

These observations are consistent with findings from a recent systematic review by Telecan et al., which reported improved learner engagement, satisfaction, spatial understanding, and academic performance with virtual dissection technologies across anatomy education. Similarly, the findings of the present review support the educational value of technology-enhanced learning interventions in neuroanatomy education, particularly in enhancing learner engagement and short-term learning outcomes. Together, the available evidence suggests that technology-enhanced learning is most effective when integrated with, rather than used as a replacement for, traditional anatomy teaching approaches [51].

Although this interventions where collectively classified as technology enhanced learning, they before considerably in their pedagogical approaches, levels of learner interaction, cognitive demand, and implementation strategies. Virtual reality interventions primarily focused on immersive experimental learning, whereas augmented reality tools integrated digital information into physical learning environments. Three-dimensional visualisation performs emphasised spatial manipulation and anatomical exploration, while stereoscopic learning systems aimed to enhance depth perception and spatial reasoning. These differences suggest that that the educational mechanism underpinning each technology may not be directly comparable. Consequently, the heterogeneity of interventions, teaching strategies, and outcome measures limits direct comparison across studies and restricts the ability to draw broad conclusions regarding the relative effectiveness of specific technology-enhanced learning approaches in neuroanatomy education.

Overall, learner motivation (L1) emerged as a critical driver of cognitive engagement. Studies such as those by Shao et al. and Allen et al. suggest that tools fostering interest, satisfaction, and interactivity can enhance knowledge retention and reduce cognitive load. Thus, well-designed digital modalities not only engage students but also support deeper learning—particularly important in a spatially demanding field such as neuroanatomy. However, technology-enhanced learning interventions were associated with increased learner engagement, motivation, and spatial understanding, improvements in objective knowledge outcomes were less consistent across studies. While several investigations reported significant gains in assessment performance, others demonstrated comparable learning outcomes between technology-enhanced and conventional teaching approaches. This suggests that the educational value of immersive technologies may extend beyond knowledge acquisition alone, particularly through their ability to enhance learner engagement and facilitate visualisation of complex neuroanatomical structures.

While most included studies demonstrated positive outcomes at Kirkpatrick Levels 1 (reaction) and 2 (learning), evidence regarding higher-level educational outcomes remains limited. Most investigations assessed learner satisfaction, motivation, and immediate post-intervention knowledge acquisition, whereas few evaluated behavioural change (Level 3) and none comprehensively assessed broader educational or clinical impact (Level 4). Consequently, the current evidence primarily reflects short-term educational benefits rather than sustained learning outcomes. Improvements observed immediately following exposure to technology-enhanced learning interventions may not necessarily translate into long-term knowledge retention, clinical competence, or application in real-world healthcare settings.

It was observed that this limitation is particularly relevant in neuroanatomy education, where the goal extends beyond examination performance to the ability to apply anatomical knowledge in clinical reasoning, diagnosis, and patient care. However studies such as Shao et al. and Faria et al. reported improvements in practical performance and anatomical identification tasks, these findings were measured shortly after the intervention and provide limited insight into whether educational gains are maintained over time. Therefore, conclusions regarding the real-world applicability and sustained effectiveness of technology-enhanced learning interventions should be interpreted cautiously.

**Fig. 3:**
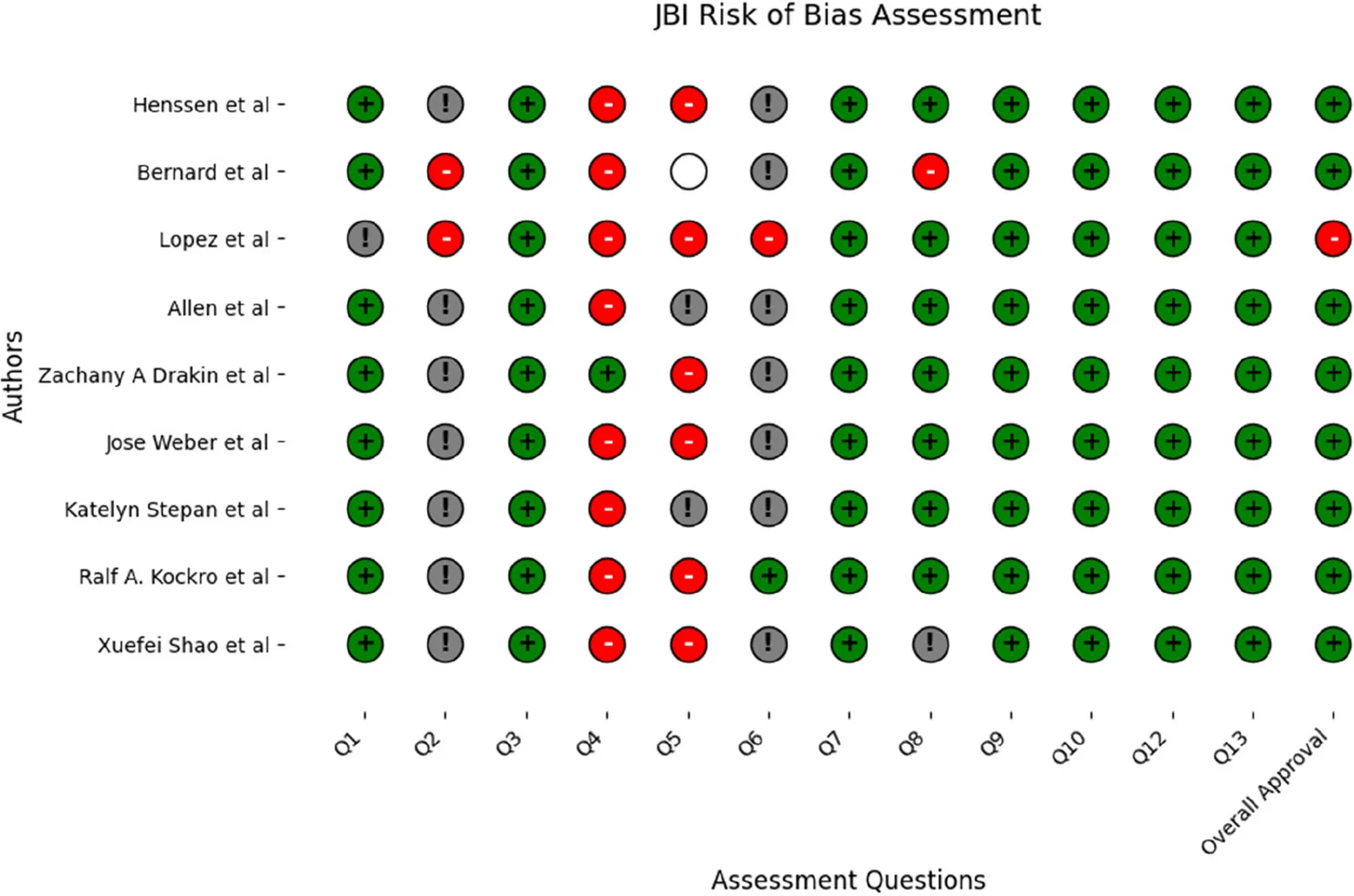
Pictorial representation of risk of bias which is described in Table 2: JBI Risk of Bias Checklist assessment (Q1-Q13). The table assesses study quality using the JBI checklist, with questions covering key domains such as randomization (Q1), allocation concealment (Q2), baseline group similarity (Q3), blinding of participants (Q4), blinding of those delivering treatment (Q5), blinding of outcome assessors (Q6), identical treatment of groups except for the intervention (Q7), completeness of follow-up (Q8), analysis of participants in the groups they were randomized to (Q9), consistency in outcome measurement (Q10), reliability of outcome measures (Q11), appropriateness of statistical analysis (Q12), and suitability of trial design (Q13). The studies are rated based on these criteria, with scores reflecting moderate to high methodological quality

The JBI Risk of Bias Checklist evaluates multiple important dimensions for Randomised Controlled Trials. This evaluation offers a detailed examination of both the methodological strength and potential sources of bias in the studies. Research studies scored between 50 and 79% which shows they possess moderate to high quality, moderate to high quality. Each study incorporated randomisation procedures to guarantee participant assignment to their respective groups. Participants were distributed between control and intervention groups using techniques that reduced selection bias. Randomisation plays an essential role in RCTs because it ensures that observed group differences originate from the treatment itself rather than other factors. Figure 3 demonstrates that all observed differences between groups resulted solely from the intervention without influence from external factors. However, the allocation concealment functions as a safeguard to keep researchers and participants unaware of group assignments beforehand. The process of concealing group assignments before the trials took place remained undefined or missing in numerous studies. In studies where allocation concealment is not well-established, there is a risk that foreknowledge of the group assignments could introduce bias into the selection or treatment processes, potentially influencing the study outcomes. Xuefei Shao et al. (2015) and Bernard et al. (2020) did not clearly report whether allocation was concealed. This may be challenging because lack of concealment can lead to a selection bias, where participants might be knowingly or unknowingly placed into groups based on their characteristics, affecting the validity of the results. Blinding was another area of concern across many studies. Blinding refers to keeping participants, those who are delivering the intervention, and outcome assessors unaware of the fact that which participant is receiving the intervention and who is in the control group. In education-based interventions, blinding participants is often difficult, as the nature of the learning tools (e.g., 3D visualisation, VR) makes it obvious which intervention group a participant belongs to. As a result, only a few studies attempted blinding of outcome assessors or blinding of treatment delivery personnel. Ralf A. Kockro et al. (2015) and Allen et al**.** (2016) did not blind participants or deliverers, increasing the potential for performance bias, where participants’ knowledge of the intervention may influence their behaviour or responses. Similarly, studies like Lopez et al. (2022) showed no efforts to blind participants, outcome assessors, or those delivering the treatment, resulting in the lowest score (50%) on the risk of bias scale. In contrast, completeness of follow-up was well-maintained in all studies. This dimension considers if all the participants that were randomly allocated into groups completed the study and whether any losses to follow-up were described and accounted for in the analysis. No missing data means confidence in the results as they are exactly what is received that will be reported. Completeness across studies minimises attrition bias, in which participants leave the study, and their motivations may affect the findings. The studies had consistent outcome measures—they all used validated assessment tools to evaluate the students' knowledge acquisition and retention. The studies all utilized validated assessments (e.g., practical tests, multiple-choice questions) to assess whether the intervention was effective in influencing results. Katelyn Stepan et al. (2017) and Jose Weber et al. (2023) both used validated questionnaires to examine knowledge in neuroanatomy, spatial cognition, and clinical judgment, adding to the rigor of the findings.

Furthermore, most of the studies used appropriate statistical analyses, which enabled sufficient detection of differences between intervention and control groups [50]. The consistency of outcome measurement across investigation has assured that the differences in student performance that we have observed are attributable to the interventions we employed and not due to measurement error or extraneous variables. The trial design across all studies was appropriate, with deviations from the typical RCT model (e.g. no blinding) noted and considered in the analysis in Zachany A. Drakin et al. (2015) and Allen et al. (2016), implementing strategies to justify appropriate handling of the randomisation and follow up processes, although blinding was not possible. These studies scored high in trial design (around 79%), reflecting a strong methodological foundation, even if some minor biases remained. Most studies were able to provide reliable outcome measurements, supporting the conclusion that technology-enhanced learning tools, especially 3D visualisation and virtual reality, can effectively enhance neuroanatomy education [52].

When interpreting the findings of this review, it is important to consider the substantial heterogeneity across the included randomised controlled trials. Although all studies employed randomisation, considerable variation existed in intervention design, implementation strategies, educational settings, and outcome assessment methods. The interventions ranged from virtual reality and augmented reality applications to three-dimensional visualisation modules and stereoscopic learning systems. Furthermore, exposure periods varied from single-session interventions to course-integrated learning experiences. Comparator groups also differed, including traditional lectures, cadaveric dissection, atlases, and conventional teaching approaches. Such variation in intervention structure and learning context limits direct comparison between studies and may partly explain inconsistencies in reported educational outcomes.

Blinding represented an additional methodological challenge. Due to the visible nature of technology-enhanced learning interventions, participants were often aware of their group allocation, making complete blinding difficult to achieve. Similarly, blinding of instructors and outcome assessors was inconsistently reported. These factors may have introduced performance and detection bias, particularly for subjective outcomes such as learner satisfaction, engagement, and perceived effectiveness. Furthermore, differences in educational environments, including classroom-based instruction, laboratory settings, and self-directed learning contexts, may have influenced learner experiences and outcomes independently of the intervention itself. Therefore, while the overall findings support the educational value of technology-enhanced learning in neuroanatomy, the observed heterogeneity should be considered when interpreting the magnitude and consistency of reported benefits.

All nine studies found evidence of intervention effects, demonstrating the effectiveness of technology-enhanced learning (TL) tools for neuroanatomy education. However, only three studies (Stepan et al., Henssen et al., Lopez et al.) included a longitudinal component, tracking outcomes beyond immediate post-tests. Additionally, subgroup analyses were present in most studies, though two did not incorporate this (Allen et al., Bernard et al.). Only two studies, Shao et al. and Henssen et al., included qualitative process evaluations, restricting deeper understanding of participants’ experiences. All studies referenced aspects of theoretical frameworks, and most presented the findings as a basis to reflect on the implications for educational theory, reinforcing their contribution toward progressing knowledge of pedagogy. The studies addressed limitations to generalisation, recognising limitations in extrapolating findings to wider educational contexts. The continued incorporation of theory and consideration of limitations strengthens the contribution of these RCTs to the field of neuroanatomy education [1].

## Conclusion

Technology enhanced learning interventions were associated with improved learner engagement, motivation, confidence, and special understanding in neuroanatomy education. 3D visualisation and virtual reality tools provided interactive learning environments that facilitate the comprehension and exploration of complex neuronal structures.

While several studies reported improvements in objective neuroanatomy learning outcomes, the evidence was not sufficiently consistent to conclude that the technology-enhanced learning is universally superior to conventional teaching approaches for knowledge acquisition. The benefit of these interventions were more consistently observed in engagement-related outcomes than in objective measures of academic performance.

The included studies demonstrated considerable heterogeneity in intervention types, pedagogical approaches, implementation strategies, and outcome measures, which limits the ability to draw generalised conclusions. Furthermore, most studies focused on short-term outcomes, with limited evaluation of behavioural change, long-term knowledge retention, or real-world educational impact. The restriction of this review to randomised controlled trials and the predominance medical student populations may further limit the generalizability of the findings to other learner groups and educational settings.

Overall, the current evidence supports the integration of technology-enhanced learning as a complimentary adjacent to conventional neuroanatomy education rather than a replacement for established teaching methods. Future research should focus on standardised outcome measures, longitudinal evaluation, and broader learning populations to better understand the sustainable impact of teaching enhanced learning in neuroanatomy education.

## Limitations

The decision to include only randomised controlled trials strengthened the methodological rigor of this review and enhanced confidence in the reported effectiveness outcomes. However, this approach may have limited the breadth of perspectives captured. Observational, quasi-experimental, and qualitative studies frequently provide valuable insights into implementation processes, learner experiences, faculty perspectives, usability, accessibility, and contextual factors that influence the success of technology-enhanced learning interventions. Consequently, while the present review provides robust evidence regarding educational effectiveness, it may not fully capture the practical and contextual considerations that influence the adoption and sustainability of these technologies within diverse educational settings.

## Supplementary Information

Supplementary Material 1.

Supplementary Material 2.

## Data Availability

Data is provided within the manuscript or supplementary information files.
