# Supplementary material for: Effectiveness of technology-enhanced learning in neuroanatomy education among health professionals - a systematic review
Source: BMC Med Educ. 2026 Jun 23;26:1425. doi: 10.1186/s12909-026-09764-w (PMC13543415; doi:10.1186/s12909-026-09764-w)
Supplement: Supplementary file 1 [file 12909_2026_9764_MOESM1_ESM.pdf]

Annexure 1

Search string for a "Effectiveness of Technology-enhanced Learning in Neuroanatomy Education among Health Professionals -A Systematic review"

Sl.no	Query for PubMed Medicine	Count
1	("Distance Education" OR "Correspondence Courses" OR "Correspondence Course" OR "Course" OR "Correspondence" OR "Distance Learning" OR "Learning" OR "Distance" OR "Online Learning" OR "Learning" OR "Online" OR "Online Education" OR "Education" OR "Online" OR "Online Educations") AND ("Neuroanatomies" OR "Neuranatomy" OR "Neuranatomies") AND ("Imaging, Three-Dimensional" OR "Imaging*, Three Dimensional" OR "Three-Dimensional Imaging*" OR "3-D Imaging" OR "3 D Imaging*" OR "Imaging*, 3-D" OR "Computer-Assisted Three-Dimensional Imaging*" OR "Computer-Generated 3D Imaging" OR "3D Imaging*, Computer-Generated" OR "Computer Generated 3D Imaging*" OR "Imaging*, Computer-Generated 3D" OR "Three-Dimensional Imaging, Computer Generated" OR "3-D Image" OR "3 D Image" OR "3-D Images" OR "Image*, 3-D" OR "Three-Dimensional Image" OR "Image*, Three-Dimensional" OR "Three Dimensional Image*")	72
2	Query for SCOPUS	
	("Distance Education" OR "Correspondence Courses" OR "Correspondence Course" OR "Course" OR "Correspondence" OR "Distance Learning" OR "Learning" OR "Distance" OR "Online Learning" OR "Learning" OR "Online" OR "Online Education" OR "Education" OR "Online" OR "Online Educations") AND ("Neuroanatomies" OR "Neuranatomy" OR "Neuranatomies") AND ("Imaging, Three-Dimensional" OR "Imaging*, Three Dimensional" OR "Three-Dimensional Imaging*" OR "3-D Imaging" OR "3 D Imaging*" OR "Imaging*, 3-D" OR "Computer-Assisted Three-Dimensional Imaging*" OR "Computer-Generated 3D Imaging" OR "3D Imaging*, Computer-Generated" OR "Computer Generated 3D Imaging*" OR "Imaging*, Computer-Generated 3D" OR "Three-Dimensional Imaging, Computer Generated" OR "3-D Image" OR "3 D Image" OR "3-D Images" OR "Image*, 3-D" OR "Three-Dimensional Image" OR "Image*, Three-Dimensional" OR "Three Dimensional Image*")	122
3	Query for CINAHL (via EBSCO)	
	("Distance Education" OR "Correspondence Courses" OR "Correspondence Course" OR Course OR Correspondence OR "Distance Learning" OR Learning OR Distance OR "Online Learning" OR Learning OR Online OR "Online Education" OR Education OR Online OR "Online Educations") AND (Neuroanatomies OR Neuranatomy OR Neuranatomies) AND ("Imaging, Three-Dimensional" OR "Imaging*, Three Dimensional" OR "Three-Dimensional Imaging*" OR "3-D Imaging" OR "3 D Imaging*" OR "Imaging*, 3-D" OR "Computer-Assisted Three-Dimensional Imaging*" OR "Computer-Generated 3D Imaging" OR "3D Imaging*, Computer-Generated" OR "Computer Generated 3D Imaging*" OR "Imaging*, Computer-Generated 3D" OR "Three-Dimensional Imaging, Computer Generated" OR "3-D Image" OR "3 D Image" OR "3-D Images" OR "Image*, 3-D" OR "Three-Dimensional Image" OR "Image*, Three-Dimensional" OR "Three Dimensional Image*")	30

4	Query for Cochrane Library (via Wiley)	
	("Distance Education" OR "Correspondence Courses" OR "Correspondence Course" OR Course OR Correspondence OR "Distance Learning" OR Learning OR Distance OR "Online Learning" OR Learning OR Online OR "Online Education" OR Education OR Online OR "Online Educations") AND (Neuroanatomies OR Neuranatomy OR Neuranatomies) AND ("Imaging, Three-Dimensional" OR (Imaging*, NEAR/2 "Three Dimensional") OR ("Three-Dimensional" NEAR/2 Imaging*) OR "3-D Imaging" OR ("3 D" NEAR/2 Imaging*) OR (Imaging*, NEAR/2 "3-D") OR ("Computer-Assisted Three-Dimensional" NEAR/2 Imaging*) OR "Computer-Generated 3D Imaging" OR ("3D" NEAR/2 Imaging*, NEAR/2 "Computer-Generated") OR ("Computer Generated 3D" NEAR/2 Imaging*) OR (Imaging*, NEAR/2 "Computer-Generated 3D") OR "Three-Dimensional Imaging, Computer Generated" OR "3-D Image" OR "3 D Image" OR "3-D Images" OR (Image*, NEAR/2 "3-D") OR "Three-Dimensional Image" OR (Image*, NEAR/2 "Three-Dimensional") OR ("Three Dimensional" NEAR/2 Image*))	03
5	Query for Web of Science	
	("Distance Education" OR "Correspondence Courses" OR "Correspondence Course" OR Course OR Correspondence OR "Distance Learning" OR Learning OR Distance OR "Online Learning" OR Learning OR Online OR "Online Education" OR Education OR Online OR "Online Educations") AND (Neuroanatomies OR Neuranatomy OR Neuranatomies) AND ("Imaging, Three-Dimensional" OR "Imaging*, Three Dimensional" OR "Three-Dimensional Imaging*" OR "3-D Imaging" OR "3 D Imaging*" OR "Imaging*, 3-D" OR "Computer-Assisted Three-Dimensional Imaging*" OR "Computer-Generated 3D Imaging" OR "3D Imaging*, Computer-Generated" OR "Computer Generated 3D Imaging*" OR "Imaging*, Computer-Generated 3D" OR "Three-Dimensional Imaging, Computer Generated" OR "3-D Image" OR "3 D Image" OR "3-D Images" OR "Image*, 3-D" OR "Three-Dimensional Image" OR "Image*, Three-Dimensional" OR "Three Dimensional Image*")	10
	Total	215

Filter used: for 10 years i.e, 2013 -2023
