# Supplementary material for: Effectiveness of technology-enhanced learning in neuroanatomy education among health professionals - a systematic review
Source: BMC Med Educ. 2026 Jun 23;26:1425. doi: 10.1186/s12909-026-09764-w (PMC13543415; doi:10.1186/s12909-026-09764-w)
Supplement: Supplementary file 2 [file 12909_2026_9764_MOESM2_ESM.pdf]

Annexure 2:

Reasons for Exclusion of Studies

During the screening process for our systematic review on the effectiveness of online learning in neuroanatomy education, studies were excluded based on specific criteria. The reasons for exclusion and the corresponding number of studies are listed below:

Primary Exclusion Criteria

- Wrong population (n = 71)
- Wrong study design (n = 60)
- Wrong outcome (n = 40)
- Not focused on online learning (n = 11)
- Review articles (n = 7)

Neuroanatomy-Specific Exclusion Reasons

- Not focused on neuroanatomy (n = 4)
- More focused on MRI studies (n = 1)
- Extra cranial and facial nerve focus (n = 1)
- Not a neuroanatomy-related study (n = 1)

Publication Type Exclusions

- Letter to the editor (n = 2)
- Background article (n = 1)

Methodological and Content Exclusions

- No abstract available (n = 1)
- No methodology described (n = 1)
- Not based on an online curriculum (n = 2)

Topic-Specific Exclusions

- Study aimed to describe the topographic relationship between craniometric points and white matter tracts of the temporal lobe through cadaveric dissection (n = 1)

These exclusion criteria ensured that only studies meeting the predefined eligibility conditions were included in the systematic review.
